# (Im)maturity in Tumor Ecosystem

**DOI:** 10.3389/fonc.2021.813897

**Published:** 2022-01-25

**Authors:** Keywan Mortezaee, Jamal Majidpoor

**Affiliations:** ^1^ Department of Anatomy, School of Medicine, Kurdistan University of Medical Sciences, Sanandaj, Iran; ^2^ Department of Anatomy, School of Medicine, Infectious Disease Research Center, Gonabad University of Medical Sciences, Gonabad, Iran

**Keywords:** stemness, immunity, immaturity, natural killer (NK), myeloid-derived suppressor cell (MDSC), dendritic cell (DC), programmed death-ligand 1 (PD-L1), immune checkpoint

## Abstract

Tumors have special features that make them distinct from their normal counterparts. Immature cells in a tumor mass and their critical contributions to the tumorigenesis will open new windows toward cancer therapy. Incomplete cellular development brings versatile and unique functionality in the cellular tumor ecosystem, such as what is seen for highly potential embryonic cells. There is evidence that maturation of certain types of cells in this ecosystem can recover the sensitivity of the tumor. Therefore, understanding more about the mechanisms that contributed to this immaturity will render new therapeutic approaches in cancer therapy. Targeting such mechanisms can be exploited as a supplementary to the current immunotherapeutic treatment schedules, such as immune checkpoint inhibitor (ICI) therapy. The key focus of this review is to discuss the impact of (im)maturity in cellular tumor ecosystems on cancer progression, focusing mainly on immaturity in the immune cell compartment of the tumor, as well as on the stemness of tumor cells.

## Highlights

- Tumors are at the interface of embryonic progeny and terminally differentiated body organs.
- Immaturity in immune ecosystem gives tumors extra potentials.
- Immature immune cells can be targeted as a supplement to the ICI therapy.
- Stemness, hypoxia, vascular abnormality, and DDR are inter-related events in tumors.
- VEGF, TGF-β, PD-L1, and EMT are mediators of immaturity.
- ATRA, GM-CSF, and TRAIL-R2 are mediators of maturity.

## 1 Introduction

**Figure 1:**
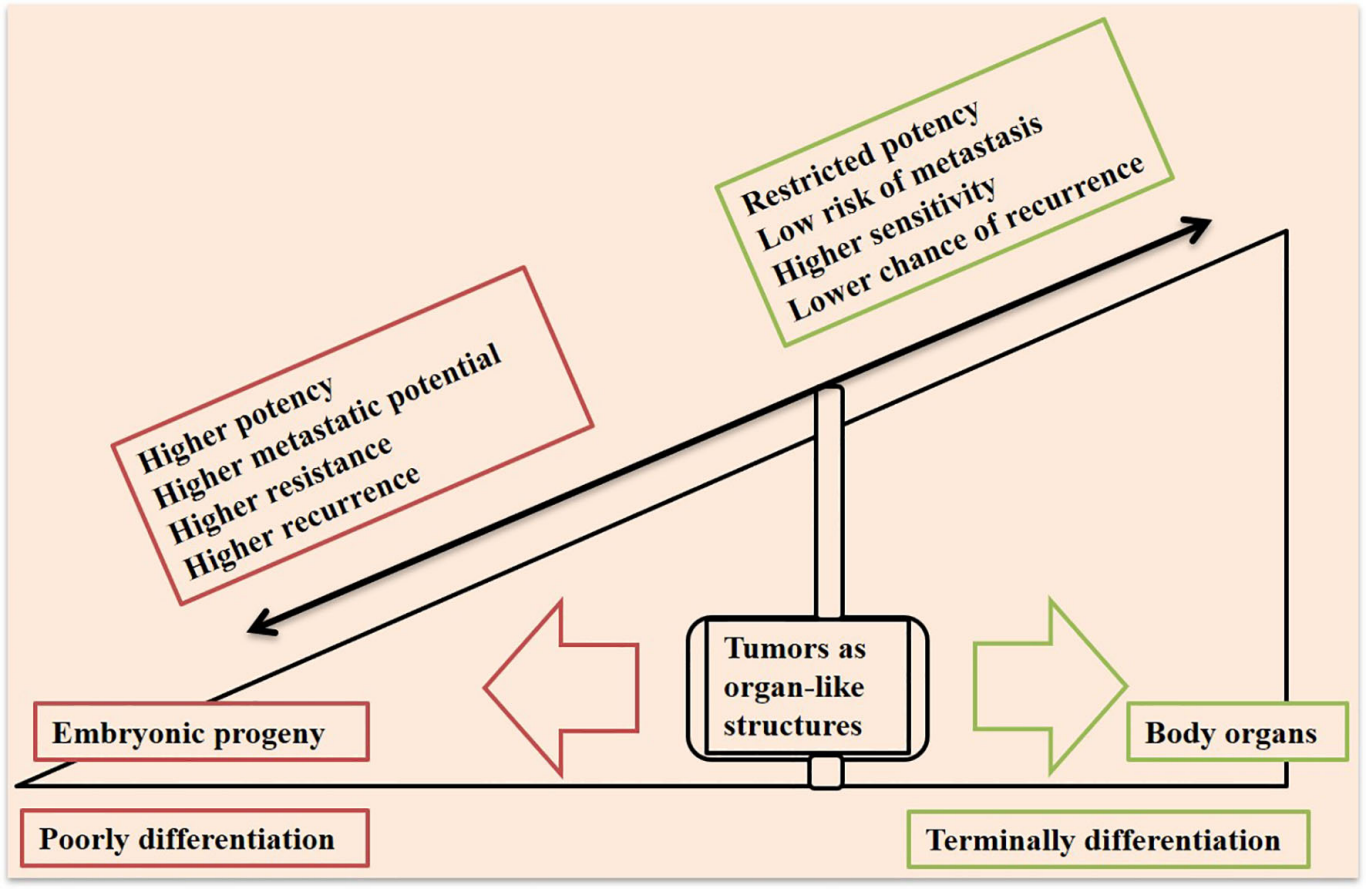
Tumors at the interface of a poorly differentiated embryonic progeny and terminally differentiated body organs. Tumors are organ-like structures that represent a pack of immature cell types, which bring them higher capacities to promote resistance, relapse, and metastasis. A tumor closer to the embryonic progeny displays higher progressive potency, whereas a more differentiated cellular state in a tumor will bring higher sensitivity to therapy.

Cancer is among the most prevalent disease in humans, and has contributed to the death of millions of people around the world (1). Solid cancers include about 80% of all human tumors and account for around 85% of cancer-related death worldwide (2). Cancer recurrence and metastasis are life threatening challenges in the area of cancer and therapy. Technology has brought new advances in the field, but there are still an outstanding number of patients who died from metastasis due to lack of efficient therapies (3). Tumors are organ-like structures that are highly ‘adaptive’ (4) and representing a rogue arena of complex and dynamic pack of cells which are heterogeneous spatially (4). Adaptation and heterogeneity give tumors extra potential, which is in contrast with normal cells in an organ working together to pursue one or more but limited directions. This infers that tumors take an evolutionary route but this is misleading as the processes are deemed ‘normal’. Aberrant angiogenesis in tumors is an example in this context, which forms vessels that are leaky, tortuous, and blind-ended with diverse diameters (5). Control over the whole organ cellularity is highly preserved in normal tissue organs. Immature or multi-potential cells in a normal organ are served to replenish organ cellularity in a pre-defined time or at the time of tissue damage and reconstruction, which is for preserving the entire tissue in homeostasis. Cells with such potential in a tumor due to bypassing growth control mechanisms will tend to destruct the cellular harmony, which finally causes more aggressive behavior. Relations between immaturity with cellular behavior within a tumor ecosystem is reminiscent of which cells are of embryonic progeny and aggressive behavior of cancer is proven (6), as it is depicted in the schematic in **Figure 1**. It has found that a well-differentiated phenotype of gastric cancer will take a G1 + G2 stage, whereas an immature cancer will take a G3 + G4 stage, which will rationalize the importance of a poorly differentiated state in high stage cancers (7).

Tumor microenvironment (TME) contains several cells that are under the control of signals and conditions within this milieu. Malignant cells stimulate stromal and immune cells to release inflammatory mediators for promoting a chronically inflamed state within TME (8). Hypoxia is a well-known condition of TME in solid cancers, which plays important roles for tumor progression (9). Conditions and signaling in TME of an aggressive tumor are acting mainly for suppression of anti-tumor immune cells, such as CD8^+^ T, macrophage type 1 (M1), and natural killer (NK) cells, while promoting the activity of pro-tumor immune cells including regulatory T cells (Tregs), myeloid-derived suppressor cells (MDSCs), and macrophage type 2 (M2) cells. In fact, tumor cells through negative interactions with anti-tumor immune cells and positive cross-talking with pro-tumor immune cells will take control over the tumor ecosystem favoring resistance and metastasis (10, 11). The key direction of this literature is to discuss (im)maturity in the cellular immune ecosystem along with the stemness of the tumor, its underlying mechanisms, and its importance in the area of therapy.

## 2 Poorly Differentiated State in Cellular Immune Ecosystem

### 2.1 Dendritic Cells

Dendritic cells (DCs) are antigen-presenting cells (APCs) that take important roles for triggering and amplifying responses from both innate and adaptive immunity against cancer (12). DCs have three subsets based on differentiation state: immature, semi-mature, and fully mature (13). In the human body, most DCs are immature and express low rates of adhesion and co-stimulatory factors. Upon stimulation by antigens, the immature cells will become differentiated into mature DCs, evolving high expressions of adhesion and co-stimulatory factors (14). DCs take key roles for induction of CD8^+^ T cell effector function. CD8^+^ T cells under exposure to the DCs will recognize major histocompatibility complex-1 (MHC-1)-bounded antigens, expressed on target cells. Thus, such effector T cells will take cytotoxic action against target cells (15). A point here is that CD8^+^ T cells will become active when they are under exposure to the ‘mature’ DCs migrated into the tumor area (16). It was found that CD4^+^ T cells upon interaction with DCs induce DC maturation (17). Mature DCs, in turn, send co-stimulatory signals in order to activate T cells (18). DC inducible effect on CD4^+^ T cell differentiation is suppressed by Tregs, depletion of which will drive anti-tumor potential conventional CD4^+^ T cells (19).

Tumor cells send signals in order to induce an ‘immature’ state in DCs (20). Interleukin (IL)-6 signaling deviates differentiation of myeloid progenitor cells from DCs into pro-tumor macrophages or taking an MDSC fate (21). IL-6 and transforming growth factor (TGF)-β (in particular) are among the main factors released from tumors at a progressive stage that act for upregulation of inhibitor of differentiation 1 (Id1) in bone marrow-derived myeloid cells. Id1 is responsible for shifting DC differentiation toward MDSCs. VEGFR is a downstream mediator of Id1 that its activity is possibly associated with hampering DC maturation mediated by Id1 (22). IL-10 is another cytokine acting primarily for blocking DC maturation (14). This cytokine is released from MDSCs (23) and M2 cells (13, 24). IL-10 also acts for activation of Tregs (23). Tregs send signals to suppress maturation of APCs (25), and the immature DCs stimulate the proliferation of Tregs (20). By contrast, maturation of DCs is promoted by CD40L (also called CD154) (26, 27). The activity of signal transducer and activator of transcriptions (STATs) is important for DC differentiation. STAT1 and STAT6 take opposing functions on DC differentiation. The activity of STAT1 is most pronounced during the maturation step, whereas STAT6 is activated constitutively in immature DCs (28).

Immature DCs promote a hypo-active state in CD8^+^ T cells called T cell anergy (29). T cell anergy is, in fact, hindering cytotoxic T lymphocyte (CTL) activation, the outcome of which is tumor progression (20). Alexia and colleagues in a study evaluated the role of polyoxidonium^®^ (PO) in breast cancer immunity. PO incubation positively influenced maturation of DCs. Such mature cells are found to display a rise in the number of co-stimulatory receptors implicated in T cell priming and CTL responses (30). In animal models, the anti-tumor efficacy of activated DCs injected inside the tumor is higher compared with the administration of immature DCs. In humans, safety and efficacy of activated DCs was evaluated in phase 1 of patients with solid cancers. Such a therapeutic approach was well-tolerated without posing dose-limiting toxicities. Infiltration of lymphocytes was found in 54% of cases. Such therapy also correlated with increased production of cytokines related with increased overall survival (31).

Immature DCs also occur in the context of infectious diseases, and their presence is contributed to less effective T cell priming (32). Lipid-based nanoparticle vaccine platform (NVP) is a strategy that can be designed to present antigens specific to the pathogenic agents, and thereby stimulating antigen-specific antibodies within the body. Such NVPs can be taken up by DCs, and are important for promoting their maturation and enhancing antigen-presenting activity against that pathogen (33). It seems that the same approach can also be designed for enhancing the maturity of these cells against tumor cells, mediated through loading NVPs with cancer cell-related antigens. Cryo-thermal therapy is another strategy that can be used for promoting a durable anti-cancer immunity. Such strategy is reported to promote DC maturation and strengthen their functionality along with expanding the proportion of other anti-tumor immune cells including M1, Th1, and CD8^+^ T cells (13).

### 2.2 Myeloid-Derived Suppressor Cells

Myeloid progeny is referred to as immature cells that upon intruding the peripheral blood will form mature macrophages, DCs, and granulocytes/neutrophils (34). Macrophages are one of the leaders of tumor immunity, representing over 50% of infiltrated cells into the stroma of the tumor. The cells take either tumor suppressor (M1) or tumor promoter (M2) phenotype in a context dependent manner (35). Fully mature DCs prime peripheral blood lymphocytes in order to form active proliferating T cells (36).

Differentiation of immature myeloid cells is impaired in chronic infections, cancer-related chronic inflammatory conditions, and autoimmune diseases, which will lead to the accumulation of MDSCs (37). An increase in the number of immature myeloid cells is prospected in tumors due to tumor tendency for surpassing immune controllers. A surge in the number of factors released from tumors into the TME interferes with normal differentiation of such cells, an outcome of which is a rise in the number of MDSCs (23, 34). Loss of maturation signals in monocytes and neutrophils will result in the accumulation of MDSCs. Immunosuppressive signals from MDSCs work against T cell infiltration and activation (10, 11, 38). By contrast, differentiation of MDSCs into APCs can introduce a therapeutic approach, mediated *via* subverting the suppressive tumor immunity (39). Suppressing MDSC maintenance and inducing their differentiation profile can be a strategy for enhancing the efficacy of ICI (40).

It seems that the suppressive activity of MDSCs is irrespective of the developmental state. Early-stage MDSCs, for instance, are a highly immature subset but show no suppressive effect on the activity of T cells. By contrast, the more differentiated granulocyte subtype (i.e., G-MDSCs) represents high suppressive activity in patients with head and neck cancer (41). Reduction of circulatory MDSC fraction is considered as a marker of more potent immunological response in cancers like pancreas (42), and is associated with improved complete response in ovarian cancer patients (43). Vaccination of mature DCs is a strategy to reduce circulatory MDSC fraction, and its application for patients with a number of solid cancers rendered a stable disease (44).

### 2.3 Natural Killer Cells

NK cells are highly heterogeneous cells that act as key effector cells against cancer, possessing the cytotoxic activity similar to that for CD8^+^ T cells (45). Unlike CD8^+^ T cells, the activity of NK cells is not dependent on antigen processing and presentation (46). Immature NK cells are highly proliferative and show suboptimal functionality. By contrast, mature NK cells are highly functional granular effector cells (47), which indicates that NK cells in order to exert killing functionality must be in a mature state. Finally, terminally differentiated NK cells show a hypofunctional state (48). NK cells upon activation release an array of chemokines and cytokines for promoting the recruitment and maturation of DCs. The matured DCs further modulate T cell activation and cytotoxic responses from CD8^+^ T cells (49). An example in this context is cervical cancer. Such cancer is induced by the human papillomavirus (HPV), and virus-like particles (VLPs) can be used as a vaccination approach against HPV-related cancer. According to results of a study maturation of DCs is increased by NK cells under exposure to the HPV-VLPs. This is mediated by upregulating HLA-DR and CD86 and repressing IL-10 production (50). The location whereby NK cells reside can determine the differentiation state of these cells. Generally, NK cells reside within the intestine and lymph nodes are in the immature state, whereas circulatory (or conventional) NK cells and cells localized within the lung, spleen, and bone marrow are fully differentiated (45).

#### 2.3.1 Mature and Immature Natural Killer Cell Subsets

Two subsets of NK cells exist in the blood of normal subjects: CD56^dim^ CD16^+^ cells vs. CD56^bright^ CD16^−^ cells. The CD56^dim^ CD16^+^ NK is a mature subtype, whereas CD56^brigh^ CD16^−^ is a marker of a less mature NK cell subset. The majority of NK cells within circulation are mature and represent cytotoxic activity (51). Such cells quickly mediate strong cytotoxic activates (52) for directly killing other cells without prior priming (51). By contrast, the less mature subtype is more localized to the secondary lymphoid tissues and shows immune modulatory activity (51). Inhibitory and activating receptors are usually co-expressed on mature NK cells in order to prevent autoreactivity (53). There is a rise in the number of mature NK cells (CD56^dim^ CD16^+^) found in solid cancer cases administered with NHS IL-12 (54). CD57 is a maker of NK cell maturation, which is contributed to the terminal differentiation of NK cells. CD57^+^ NK cells undertake the final maturation step from CD56^dim^ CD57^-^ into CD56^dim^ CD57^+^ cells, a phenotype that is known to be highly cytotoxic (55). High CD16 and NKG2D expressions are found in NK cells upon expansion for adoptive immunotherapy (56). In fact, antibodies engaging NK cells through biding to the activating receptors CD16 and NKp46 along with an antigen on tumor cells are considered as the next generation molecules for cancer immunotherapy (57).

#### 2.3.2 Functional Circuity Between Natural Killer Cells, Dendritic Cells, and CD8^+^ T Cells

There is a net of functional circuity between NK cells with DCs and CD8^+^ T cells, which results in a maximized effector function against cancer (58). Direct cell-to-cell contact between NK cells with DCs promotes immune responses through induction of DC maturation (11, 59). Matured DC cells can uptake tumor antigens from secondary lymph nodes. Such antigens are presented to the T cells and are contributed to their activation (60). DCs by turn act for priming NK cells in order to exert effector responses (48).

#### 2.3.3 Mature Natural Killer Cells in Cancer Immunotherapy

Allogenic administration of mature NK cells can be a desired approach for boosting immune functionality against cancer (61, 62). ‘Education’ is a term used for functional maturation of NK cells in which the educated cells are highly responsive to cells that lack the expression of self-MHC class (63). Generation of such highly functional cells is a focus of adoptive immunotherapy, and it is not restricted to the NK cells in which adoptive mature T cells can also be used in cancer cellular immunotherapy (64).

**Figure 2:**
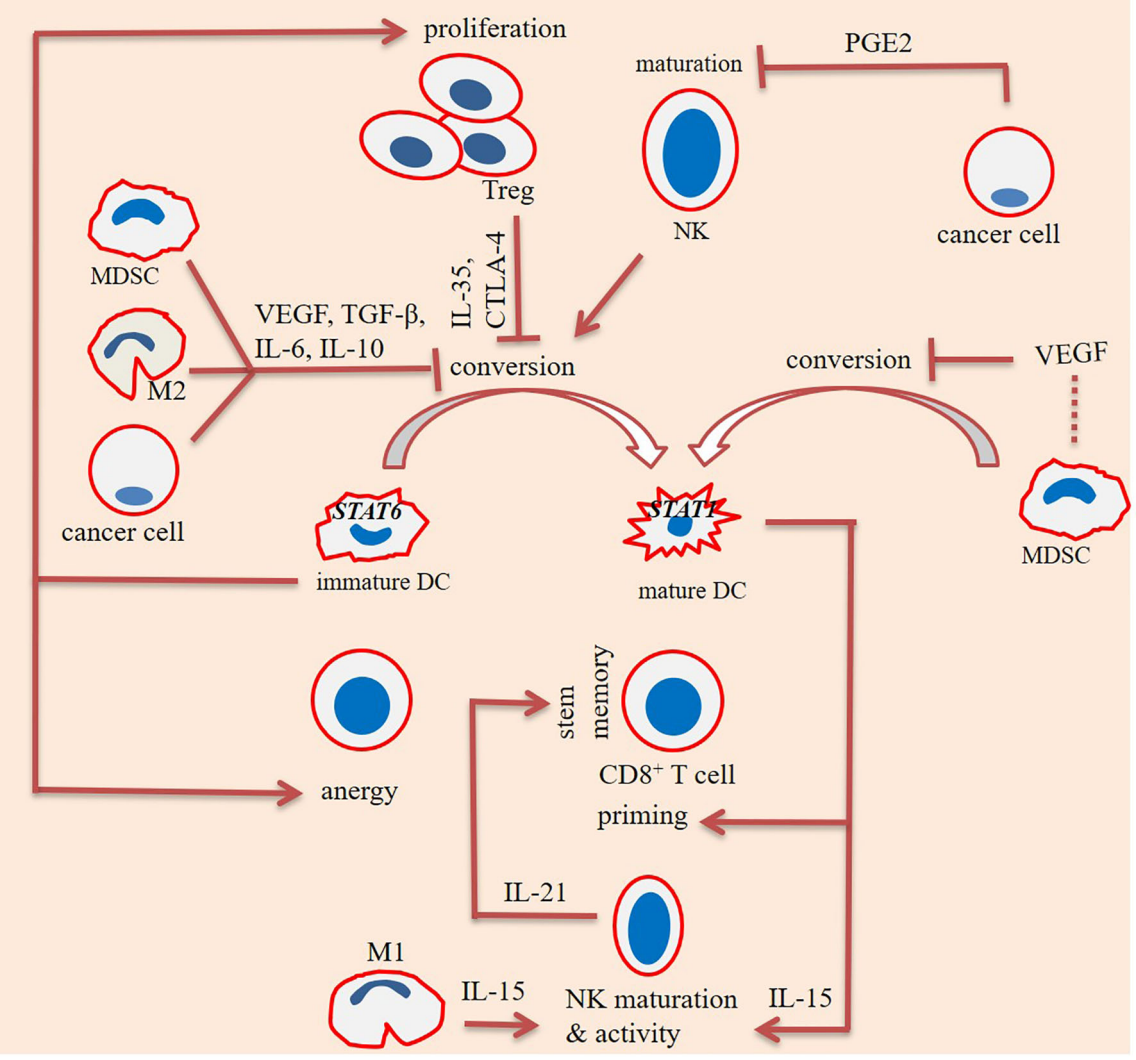
Interactions among immune cells for promoting maturity/immaturity within the tumor immune ecosystem. Maturation of dendritic cells (DCs) is affected positively from the impact of natural killer (NK) cells, but negatively affected from the effects of cancer cells, regulatory T (Treg) cells, and myeloid-derived suppressor cells (MDSCs). Interleukin-10 (IL-10), cytotoxic T lymphocyte-associated antigen-4 (CTLA-4), and vascular endothelial growth factor (VEGF) are inhibitory factors released from cancer cells, Tregs, and MDSCs, respectively. IL-10 is also released from MDSCs and macrophage type 2 (M2) cells. Tregs impede DC maturation through CTLA-4 and IL-35. Immature DCs are signal transducer and activator of transcription 6 (STAT6)^+^ and take tumor-promoting activities, mediated *via* enhancing Treg proliferation and stimulating T cell anergy. By contrast, mature DCs are STAT1^+^ and stimulate CD8^+^ T cell priming and NK cell effector function. Cancer cells release high levels of prostaglandin E2 (PGE2) upon exposure to hypoxia. PGE2 hampers maturation of NK cells. By contrast, maturation of NK cells is promoted by IL-15, released from DCs and M1 cells. NK cells further release IL-21 for promoting stem-like memory CD8^+^ T cells.

**Figure 3:**
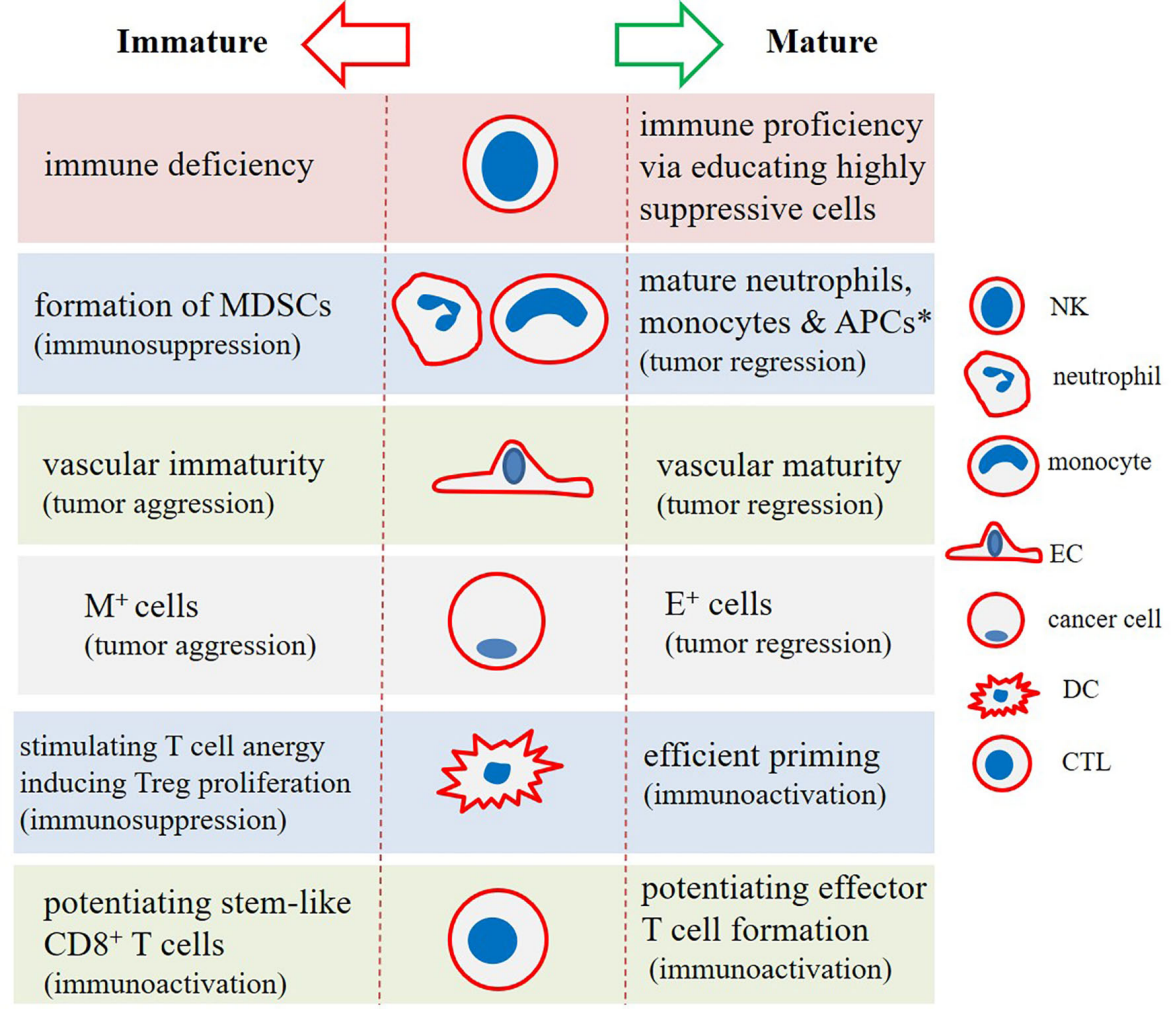
The impact of (im)maturity on final functional state in tumor immune ecosystem. Natural killer (NK) cells, dendritic cells (DCs), and cytotoxic T lymphocytes (CTLs) in a mature cellular state will strengthen immune proficiency against cancer. Stem-like immune niches exist in tumors, strengthening of which will lead to the more effective cancer immunotherapy. Monocytes and neutrophils in an immature state will form myeloid-derived suppressor cells (MDSCs), which act as strong suppressors of the immune system. Similarly, immature endothelial cells (ECs) form aberrant vasculature, which aids tumor progression and relapse. This is also inferable for mesenchymal (M^+^) tumor cells (cancer stem cells [CSC] in particular) that represent high resistance and tumor-promoting activities. This contrasts with cells with epithelial-like (E^+^) phenotype. *Antigen-presenting cells (APCs) including DCs and macrophages.

T cell immunoglobulin mucin-3 (TIM-3) is a checkpoint mediator that its expression is related to both NK cell maturity and exhaustion (60). Based on the results of one study TIM-3^+^ NK cells were higher in colorectal cancer (CRC) patients who were not developing metastasis, and that the presence of such cells was related negatively with cancer stage (65). Ndhlovu and colleagues in a study found essential expression of TIM-3 on CD56^dim^ CD16^+^ NK cells, and the link between TIM-3 with NK cell maturity. It is interesting to note that expression of TIM-3 as a checkpoint mediator on T cells is considered as a marker of cell dysfunction (66). Cytotoxic T lymphocyte-associated antigen-4 (CTLA-4) is another checkpoint that is negatively related with cytotoxic activity of NK cells. CTLA-4 inhibition using ipilimumab in human melanoma patients by Tallerico and colleagues resulted in the higher frequency of mature NK cells (CD3^−^CD56^dim^ CD16^+^) within circulation (67). Recently, Bi and colleagues reported a negative link between expression of checkpoint TIPE2 with IL-15-mediated NK cell maturation. The authors noticed enhanced maturation in the TIPE2-deficient NK cells. The cells also had higher activation and cytotoxic activities. Targeting TIPE2 can thus be an approach in NK cell-based immunotherapy (68). The maturity state among immune cells and interactions among them along with the impact of differentiation states on the final fate of cancer is illustrated in **Figures 2**, **3**, respectively.

## 3 Stem-Like Immune Niches

A study by Jansen and colleagues has shown stem-like immune niche breakdown as a potential mechanism of immune escape by tumors. These niches are the host for stem-like CD8^+^ T cells, contributed to the formation of effector T cells for targeting tumors. A point here is that the presence of these niches is required for strengthening T cell infiltration toward the tumor area. Scarcity of these immune stem niches occurring progressively after surgery along with an inefficient stimulation of cells within these niches is a proposed reason for attenuated responses from T cells against human cancers (69). The idea has also been approved in animal models of cancer in which effector T cells in a terminally differentiated state and effector memory T cells in a highly differentiated state although being able to kill tumor cells effectively, their anti-tumor potential is lower than that for less differentiated central memory T cells. As reported, such reduced anti-tumor capacity is attributed partially to the CD27 and CD62L loss of expressions in highly (or terminally) differentiated T cells. T cells express CD27 as a molecule essential for their long-term survival. T cells express the adhesion molecule CD62L for entering secondary lymphoid organs, a crucial and required step for generation of pools of long-lived memory T cells. Expression of CD62L by T cells can be a main determinant factor for enabling them to infiltrate into the tumor area (36). Therefore, promoting the development of tumor suppressive T cells in a state not high in the differentiation is important therapeutically. Maintaining stem-like state in T cells is preferentially effective before their adoptive transferring (70). Krishna and colleagues performed a study evaluating CD8^+^ T cell states as a response to the question why some patients respond effectively to adoptive cell therapy (ACT), while others do not. The authors have come with important findings in which in patients responsive to the ACT a subset of cells with stem-like features were detected. Such stem-like cells were CD39^-^CD69^-^ and low in number but were efficient to cause complete regression of cancer and to maintain the fraction of tumor-infiltrating lymphocyte (TILs). By contrast, the presence of terminally differentiated CD39^+^CD69^+^ cells was linked to the weak TIL persistence (71). Patients in the convalescent phase of infectious diseases are also found to display polyfunctional T cells and stem-like memory CD8^+^ T cells. Such early differentiated memory cells possibly bring protection against the virus (72). Therefore, preserving stem-like memory cells of CD8^+^ T progeny can offer a therapeutic opportunity in cancer patients.

The fraction of stem-like progenitor cells is expanded in patients receiving immune checkpoint inhibitors (ICIs) (73). T cell transcription factor 1 (TCF-1) is a transcription factor that is placed downstream of Wnt signaling. The activity of this transcription factor is essential for development and maturation of T cells (74). PD1^+^TCF1^+^ T cells are memory progenitor cells that can provide T effector cells, whereas PD1^+^TCF1^-^ T cells are terminally differentiated (73). TCF-1^+^TIM-3^−^ CD8^+^ T cells are progenitor exhausted and display a relatively high proliferative ability, while TCF-1^−^TIM-3^+^ CD8^+^ T cells show terminally exhausted phenotypes (75).

Exhausted CD8^+^ T cells expressing TCF1 preserve their effector function upon encountering chronic viral infection (74). However, terminally exhausted CD8^+^ T cells do not respond to ICI. Fusion protein for IL-10–Fc has found to promote metabolic reprogramming toward oxidative phosphorylation and revitalizing terminally exhausted cells more responsive to immunotherapy (75).

Stem-like memory CD8^+^ T cells is induced by IL-21, their differentiation is induced by PD-1 inhibitors (76), but their terminal differentiation is induced by IL-2 (77). Thus, IL-21 can be fused to the anti-PD-1 antibody and is an effective strategy for increasing the anti-tumor activity of tumor-specific T cells (76, 78). IL-21 fusion to anti-PD-1 antibody has found to promote generation of stem-like memory T cells with higher proliferative activities (76). It is interesting to note that H9T which is an engineered partial agonist of IL-2 can promote CD8^+^ T cell expansion without causing their terminal differentiation. Incubation with H9T caused sustained expression of TCF-1 and induced mitochondrial fitness, favoring sustained stem-like state of CD8^+^ T cells. CD8^+^ T cells exposed to H9T represent strong anti-tumor activity, as reported in a mouse model of melanoma (70).

CTLA-4 and PD-1 are inhibitory members of the CD28 family receptors (79). CTLA-4 interaction with CD80/CD86 promotes immune regulation, whereas interaction between CD28 with CD80/CD86 promotes immune activation. CD28 acts in the promotion of T cell stemness and proliferation. In fact, T cells upon maturation will lose CD28 (80). A different path occurs in DCs. CD80/CD86 is also expressed on DCs (81). Potentiating the expression of co-stimulatory molecules CD80/CD86 in DCs is positively related to their maturation. Reducing the expression of these receptors is a mechanism by which IL-35 impedes maturation of DCs (82). IL-35 is expressed by Tregs (83), and its activity is considered a hallmark of immune regulation in cancer (84). It seems that in patients receiving CTLA-4 inhibitors, such as ipilimumab, the binding sites on these receptors will be reopened for CD28 and further induction of DC maturation and activity, along with the promotion of T cell-mediated immunity against cancer.

## 4 Factors Related to the Differentiated Cellular State in Tumor Immune Ecosystem

### 4.1 Agonists of All-Trans Retinoic Acid

All-trans retinoic acid (ATRA) is a derivate of vitamin A that plays a key role in cellular proliferation, differentiation, and apoptosis (85). ATRA acts to reduce the number of MDSCs and the immunosuppressive genes expressed by these cells, such as programmed death-ligand 1 (PD-L1) and TGF-β. ARTA induces a differentiation program in MDSCs, an outcome of which is reduced MDSC fraction (42). ARTA was used by Rao and colleagues in mouse tumor models, which causes higher induction of inflammatory macrophages that resulted in lower radio-resistance (86). Administration of ATRA also overcomes chemoresistance in breast cancer (87).

### 4.2 Agonists of TNF-Related Apoptosis Induced Ligand-Receptor-2

TNF-related apoptosis induced ligand (TRAIL) is a homotrimeric protein that its main role is for modulating immune responses (88). Expression of TRAIL on T and NK cells is important for controlling tumor immune surveillance (89). TRAIL type 2 receptor (TRAIL-R2) (also called death receptor 5/DR5) is a membrane-bound death receptor (90) that is expressed mainly on endothelial cells (ECs) and immune cells (88). Selective targeting of MDSCs is applicable using TRAIL-R2 agonists. TRAIL-R2 agonists are not acting on mature myeloid and lymphoid cells. DS-8273a is a TRAIL-R2 agonist that its application in clinical trials has shown impressive effects on tumoral MDSC fraction and improving clinical outcomes, but the effects are temporal and depended on the time factor (91).

### 4.3 Granulocyte-Macrophage Colony Stimulating Factor

Granulocyte-macrophage colony stimulating factor (GM-CSF) is a growth factor of hematopoietic cells that promotes macrophage, DC, and neutrophil development. GM-CSF also induces maturation and activation of DCs and their further recruitment into the tumor area (43, 92, 93). Results of pre-clinical trials with GM-CSF were promising for rendering a more immunogenic tumor contexture (92). Clinical trials for application of GM-CSF in human cancers are numerous. In CRC, administration of GM-CSF was found to induce anti-tumor CTL responses (94). GM-CSF encoded to the herpes simplex virus in advanced melanoma patients caused a meaningful objective response rate (ORR) (28%) (95). GM-CSF cell-based vaccine (GVAX) is suggested as an appropriate combination to go with ICI therapy of pancreatic cancer (96). Finally, administration of nab-paclitaxel and GM-CSF in platinum resistant ovarian cancer patients has been found to result in an ORR of 72% (partial and complete responses in 43% and 29% of cases), but the outcomes were not durable (43). In animal models of cancer, a link between G-CSF (97) or GM-CSF (98) with increased MDSC recruitment into the tumor area were reported. There is also a report of a positive link between GM-CSF with M2 recruitment toward tumor cells of BRCA1-IRIS over expressing (IRISOE) triple-negative breast cancer (99). Such effects are possibly occurring at high GM-CSF levels, however needing more research in order to make clearer the real effect of this glycoprotein in solid tumors.

## 5 Factors Related to the Immaturity in Tumor Immune Ecosystem

### 5.1 Programmed Death-Ligand 1/Programmed Death-1 Receptor

PD-L1 is a checkpoint molecule that acts *via* interaction with programmed death-1 receptor (PD-1). The activity within PD-1/PD-L1 axis is considered as a main co-inhibitory checkpoint pathway for modulating immune evasion in cancer patients (100). PD-1 is expressed on the surface of CTLs, DCs, and NK cells, while PD-L1 is expressed by Tregs, MDSCs, M2 cells, and cancer cells, in particular (101). PD-1^+^ NK cells harvested from healthy cells were found to show a mature phenotype (CD56^dim^ phenotype) (102). A rate of PD-1 expression on anti-tumor immune cells is appropriate for their functionality. By contrast, high expression of PD-1 attracts more inhibitory ligands from TME, thus turning them into a state called dysfunctionality or exhaustion. Therefore, PD-L1 overexpression is related to the T cell dysfunctionality and is deemed as poor tumor prognosis (101, 103–105). Blocking the activity of PD-1/PD-L1 axis will restore T cell functionality, mediated through reinvigorating cancer antigen–specific T cells (106, 107). PD-1 ligation suppresses long-lived effector T cell functionality. Altered metabolic reprogramming is a mechanism by which PD-1 determines T cell differentiation (108). Monotherapy with PD-1 inhibitors will shift the metabolic profile toward glycolysis, the outcome of which is the terminal differentiation of T cells. Such cells are functional but short-lived and undergo quick apoptosis. By contrast, combination of PD-1 inhibitors with metabolic regulators, such as Bezafibrate, will increase oxidative phosphorylation, the result of which is long-lived functional T cells (109). Mature T cells also show mutations in JAK and STAT3, so that inhibitors of JAK and STAT3 will hamper PD-L1 expression (106), which is possibly indicative of a role for PD-L1 inhibitors for promoting maturation of T cells.

Blocking the activity of PD-1/PD-L1 axis will restore T cell functionality, mediated through reinvigorating cancer antigen–specific T (106, 107). Of notice, PD-L1^+^ cancers show higher responses to ICI compared with PD-L1^-^ tumors. This indicates the importance of PD-L1 as a marker of tumor response to the immunotherapy. This, however, is not effective for patients with cold immunity in which solo PD-L1 is not considered as a biomarker of response to the ICI therapy. For such patients, PD-L1 expression along with the rate of T cell infiltration and/or tumor mutational burden (TMB) is considered as a biomarker of response (101).

### 5.2 Vascular Endothelial Growth Factor

Vascular endothelial growth factor (VEGF) is a pro-angiogenic factor that is overexpressed in tumors, and its persistent hyper-release in TME promotes vascular immaturity and abnormal angiogenesis, delineated by leaky (or permeable) vessels (110). Cancer-associated fibroblasts (CAFs), M2 cells, cancer cells, and ECs are the four key sources of VEGF in TME (111). VEGF overexpression acts as a promoter of an immature vascular net (112), and that development of such immature vessels has a profound impact on tumor progression, mediated through promoting immunosuppression and therapy resistance (111). VEGF has an inhibitory effect on maturation of MDSCs into APCs (113). Maturation (or differentiation) of DCs is also precluded by VEGF (114, 115), a result of which is impaired effective T cell priming (116) and inactivation of CTLs (115). In addition, VEGF impairs the migration ability and function of mature DCs (12). Patients with microsatellite stability CRC show low baseline infiltration of T cells, and the existing cells are undergoing a dysfunctional state called exhaustion. Exhausted T cells are formed due to hyper-functionality and are ineffective to work against cancer. VEGF is a key driver of such exhaustive state (117). Exhausted CD8^+^ T cells in hypoxic conditions secrete VEGF-A, and it has found that secretion of this pro-angiogenic factor is linked to their terminally exhausted state. This is an outcome of an *in vitro* study by Bannoud and colleagues. Terminally exhausted T cells due to representing a terminal differentiation position do not recover their effector functionality using ICIs (118).

Targeting VEGF by bevacizumab in cancer patients reduces the number of immature circulatory DCs (119). VEGF-trapping approach is a strategy to inactivate VEGF within the extravascular space and in the bloodstream. Fricke and colleagues in a study used VEGF-trap for a number of advanced-stage cancer patients and found the efficacy of this strategy in improving DC maturation; however, the efficacy of this approach cannot be achieved unless reducing the number of MDSCs as well (114). VEGF targeting therapy has also been approved to be used in combination with ICI for human cancers (120). The immune suppressive effect of VEGF on APCs and immune effector cells, and the positive impact of immunosuppressive cells for driving angiogenesis create a vicious cycling of impaired immune functionality. This will justify application of anti-angiogenic therapy in combination with ICI for strengthening effector immune activity against cancer and reducing the rate of immune escape by tumor (121). A suggested strategy is to use vascular normalizing agents to go with ICI for improving the efficacy of immunotherapy (122).

### 5.3 Hypoxia and Hypoxia Inducible Factors

Hypoxia is an O_2_ low condition that is presented within TME due to higher cellular proliferation rate compared with blood supply. Hypoxic tumors are more aggressive and are more prone to developing resistance and metastasis (5, 9, 111, 123–126). Hypoxia influences the fraction of immune infiltrates and the spatial association between tumor and immune cells (127). The immunosuppressive effect of hypoxia is a barrier for efficacy of therapy in patients receiving ICI therapy. Elevated oxidative metabolism by tumor cells will lead to a rise in intra-tumoral hypoxia and a fall in the fraction of CD8^+^ T cells (128).

Hypoxia inducible factor (HIF)-1 plays a vital role in the hypoxic TME. HIF-1 inhibits the activity of innate and adaptive immunity against cancer (129). Under inflammatory conditions the activity of HIF-1α promotes the maturation of DCs and their subsequent activity (130). In tumoric conditions, by contrast, maturation of both DCs and NK cells is inhibited (129). Hypoxia suppresses differentiation of T effector cells (131). Hypoxia can be targeted as a way for retaining the effector function of NK cells (126, 132). IFN-I stimulates DC maturation, and thereby supports CTL activity. IFN pathway is downregulated by hypoxia both transcriptionally and translationally, which is a reason for hypoxia-mediated immunosuppression in tumors (133). The immature DCs upregulating HIF-1α undergo early apoptosis (134). Constitutive activation of HIF-1 in tumors at advanced stages maintains elevated levels of MDSCs (135). Increased MDSC arginase activity is promoted under exposure to HIF-1α (131). There is a report that arginase-1-expressing MDSCs infiltrated into the tumor area are predominantly immature and have a monocytic subtype (136). HIF-1 is also related positively with B cell immaturity, which will lead to a decrease in the number of mature B cells within peripheral blood (137).

In hypoxic TME lactate dehydrogenase A (LDHA) shows increased expression (as a response to the high HIF-1α activity). Upregulation of LDHA results in more lactate production and is seemingly linked with CD8^+^ T cell senescence and exhaustion. A combination of LDH inhibition along with IL-21 has been found to be a useful strategy for strengthening CD8^+^ T cell stemness (77). Hypoxic TME induces tumoral cell expression of prostaglandin (PG)-E2 (138), high presence of which suppresses maturation of NK cells (139). In hypoxic CRC, HIF-1 bonds to the glutaminase 1 (GLS1) promoter and increases the conversion of glutamine to glutamate. Such reduction in the glutamine extracellular concentration is linked to deregulated T cell differentiation, inhibiting differentiation of Th17 and Th1 whereas maintaining differentiation of Tregs (126). VEGF expression in tumor stroma is induced by hypoxia (110), which links hypoxia with aberrant angiogenesis, as discussed elsewhere (111, 140). This is indicative of the importance of vascular normalization strategies as an effective way for alleviating tumor hypoxia (141, 142). Hypoxia also regulates tool-like receptors (TLRs) for influencing maturation of immune cells. TLRs are members of pattern recognition receptors (PRRs) that take both pro- or anti-tumor activities in a context dependent manner (143). DC maturation, for instance, is promoted by TLRs (144). TLRs promote a metabolic shift toward glycolysis, which is considered as a required step for DC maturation. This will allow survival of the cells after activation (145).

### 5.4 Transforming Growth Factor-β

TGF-β is a multi-tasking cytokine that acts in an important role during development and in tumorigenesis. The activity of TGF-β in normal tissues is for promoting differentiation of epithelial cells, whereas in established tumors it acts for initiating cell self-renewal and epithelial-mesenchymal transition (EMT), namely promoting an immature state in tumoral cells (146). TGF-β activity is important for all stages of tumorigenesis (147), and its high levels is associated with immune escape and tumor metastasis (146). TGF-β can thus be a desired target in the area of cancer therapy (148).

Development and differentiation of NK cells is influenced tightly from TGF-β signaling (149). Marcoe and colleagues in a study on mouse immunity development have found a link between TGF-β activity with NK cell immaturity. TGF-β is responsible for inefficient NK cell responses early in life. By contrast, a pool of NK cells undergo maturity in the absence of this signaling (150). Hampering NK cell maturity in the presence of TGF-β will make the host more susceptible to viral infections (149) and tumor progression. Elevated levels of TGF-β also block the differentiation of naïve T cells into Th1 effector cells, instead promoting their conversion into Treg subset (151, 152). TGF-β inducible effect on Foxp3^+^ Treg expansion suppresses differentiation of Th17 cells through antagonizing RORγt (153).

**Figure 4:**
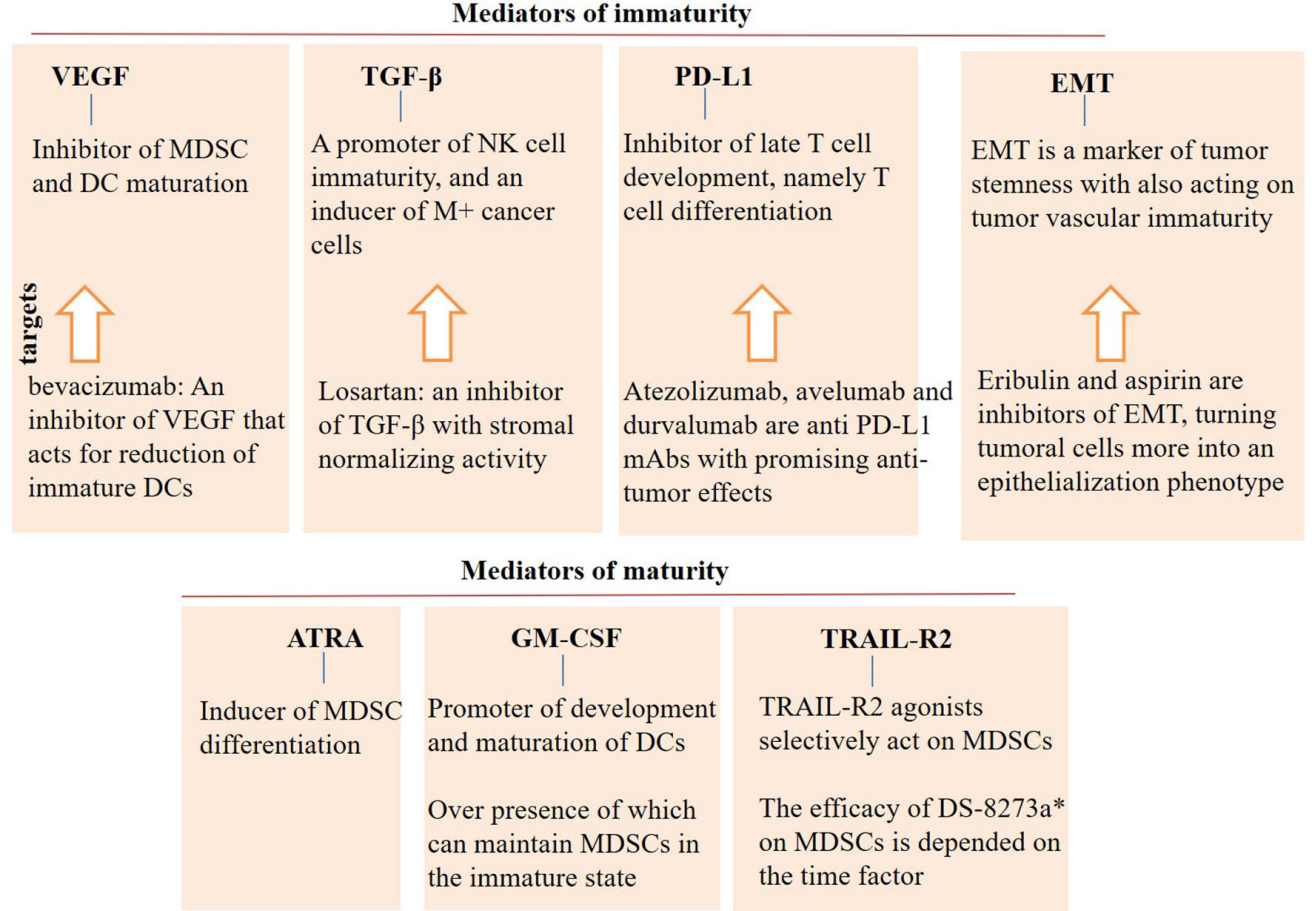
Mediators of (im)immaturity. Vascular endothelial growth factor (VEGF), transforming growth factor (TGF)-β, programmed death ligand-1 (PD-L1), and epithelial-mesenchymal transition (EMT) are mediators of immaturity that contribute to the tumor progression and relapse. By contrast, agents like all-trans retinoic acid (ATRA), granulocyte-macrophage colony stimulating factor (GM-CSF), and TNF-related apoptosis induced ligand-receptor2 (TRAIL-R2) are mediators of maturity that are contributed to cellular differentiation. Agonists of maturity mediators can be an appropriate supplement in the area of immunotherapy. Controversies, however, exist for the use of GM-CSF, which require more research in the area. DC, dendritic cell; MDSC, myeloid-derived suppressor cell; and mAb, monoclonal antibody. *DS-8273a is a TRAIL-R2 agonist.

Monocytic MDSCs are highly expanded under the influence of TGF-β. When TGF-β is presented in the area, IL-6 is the most potent stimulator of MDSC functionality, whereas G-CSF is strongly active in the absence of TGF-β (154). The efficacy of ICI is also enhanced when TGF-β signaling is blocked in the tumor area (155). To explain, TGF-β inhibitors are acting as immune modulatory agents that when used in combination with ICI, will render more effective outcomes. A summary of the factors related to the (im)maturity cellular immune states is represented in **Figure 4**.

## 6 Cancer Stem Cells

Cancer stem cells (CSCs) are cells that their number is increased at the time of tumor progression. An increase in the number of these cells will give a tumor more chance to resist and relapse (124). CSCs can acquire a transient, hybrid phenotype enabling them to have control over normal tissue nearby and to neutralize the hazardous impact of the nearby environment. It is interesting to note that even committed (differentiated) cells in healthy tissues can reinstate a feature of undifferentiated state upon encountering a harm condition. This occurs when the reservoir of stem cells is not sufficient to exert a complete response. Acute wounds occurring in the body are good examples in which a cellular differentiation state is affected in order to retain the healing potentials. Cancers are chronically wounded, which gives them extra potentials, delineated by packs of CSCs or tumor cells with stemness features (5, 156, 157).

Targeting signals of stemness is a way for controlling the fraction of CSCs within a tumor. A suggested strategy to pose durable anti-tumor responses is to recall signals of differentiation in CSCs. Ronen and colleagues in a study found loss of invasion and metastasis of breast tumor when cancer cells with EMT phenotype were transdifferentiated into post-mitotic functional (mature) adipocytes (158). Immature adipocytes are contributed to the augmentation of CSC fraction (159). Chemotherapy preferentially acts on proliferative cancer cells, so trans-differentiation of CSCs in dormancy into mature proliferative cells will allow their further elimination by chemotherapy (160). De-differentiation is a process by which tumor cells can retain stemness profile when they are under exposure to the conditions like hypoxia and is contributed to tumor relapse after therapy. Thus, a suggested strategy could be targeting mediators or promoters of de-differentiation (161).

## 7 Factors Influencing (Im)maturity of Tumor Cells

### 7.1 Aberrant Angiogenesis and Vascular Abnormality

Aberrant angiogenesis is an important step for cancer progression, which is linked to the tumor growth and metastasis (162). Aberrant tumor vessels can be a route for highly invasive tumor cells (163). Such weakly functional tumor vessels promote hypoxia and immunosuppression, thereby causing tumor progression (164). Carbonic anhydrase 9 (CA9) is a hypoxia-induced enzyme responsible for regulation of pH in hypoxic solid cancers. CA9 can be targeted in order to turn an abnormal (immature) tumor vasculature into normal (mature) vessels (165). A normal mature vasculature allows more infiltration of effector T cells, while an immature vessel restricts penetration of these anti-tumor immune cells (166). Vascular normalization is a strategy to reduce immunosuppression and CSC resistance in tumors (111). Kashiwagi and colleagues evaluated the efficacy of eribulin in metastatic breast cancer patients and noticed EMT reversion and vascular remodeling in response to this microtubule dynamic inhibitor. They noticed negative conversion of CA9 and durable responses to such therapy (165), highlighting the importance of vascular normality in tumor targeted therapies.

### 7.2 DNA Damage Response

DNA damage response (DDR) and aberrations in gens related to DDR is representative of a metastatic cancer. DDR occurs as a response to high reactive oxygen species (ROS) (not ROS overloading) in TME, and is a trigger for cancer cell reprogramming into CSCs with EMT phenotype, thereby increasing the number of therapy resistance CSCs (156). Olaparib is an inhibitor of poly(ADP-ribose) polymerase (PARP)1/PARP2, known as the key DDR-related genes. Olaparib was administered to metastatic prostate cancer patients with aberrant DDR genes. Mateo and colleagues in this study noticed an improvement in endpoint responses, delineated by low circulatory tumor cell (CTC) fraction (167). However, Zuo and colleagues in a recent study reported a positive link between Olaparib with an increase in the number of immature myeloid cells; such cells create an immunosuppressive milieu and act for augmenting the rate of metastasis (168). Therefore, myeloid-targeting agents are requested in patients receiving Olaparib.

### 7.3 Epithelial-Mesenchymal Plasticity

#### 7.3.1 Epithelial-Mesenchymal Plasticity in Tumor Cells

Epithelial-mesenchymal plasticity (EMP) is a highly flexible cellular state that is mainly presented in the context of cancer stemness and resistance (169), defining a route for cancer heterogeneity (170, 171). Carcinoma cells including CSCs will take EMT and the reversed process mesenchymal-to-epithelial transition (MET) in order to adopt the environment nearby (170) and to take invasive behavior and to promote resistance (172). Tumor cells will take MET in order to develop a macrometastasis tumor in secondary sites (171, 173). Activation of an EMT program in mammary epithelial cells will thus expand the generation of chemo- and immune-resistant CSCs (174). EMT gives the cells increased motility potential and cellular dissemination toward the circulatory system. TWIST1, SNAIL, and ZEB are transcription factors related to EMT (172). Acquiring an EMT profile is a response to active signaling, such as TGF-β (175).

Oliphant and co-workers found a direct link between upregulation of genes related to the pluripotency with late metastasis of breast tumor cells. They reported that recalling an embryonic stem program by factors like Six2 will bring higher potencies to the early-detached tumor cells (176). EMP is a capacity that is designated to the poorly differentiated cells. A study by Shinde and colleagues showed that overexpression of tissue transglutaminase-2 (TG2) in breast tumor cells is sufficient for augmenting the development of metastatic niches and promotion of distant metastasis, whereas TG2 depletion suppresses metastasis. TG2 is a gene that emerges solely in metastatic cells undergoing EMT induction/reversion. The outcome of this study represents how inter-conversions between the two different cellular states, namely epithelial and mesenchymal cells allows tumor cells to shape a metastatic fate (177). Agents targeting EMP will yield a strong and wide class of therapeutic drugs (173). Targeting EMP will increase responses to the ICI and enhance the duration of responses (170). TGF-β is contributed to the activation of SNAIL1 and induction of ZEB proteins (172), so it can be a promising target for reducing the risk of resistance and metastasis.

#### 7.3.2 Epithelial-Mesenchymal Plasticity in Circulatory Tumor Cells

A high number of CTCs is reflexive of the progressive disease, and their fraction in a tumor like breast cancer can be a reflective of the total tumor burden (178). CTCs represent monoclonal and polyclonal (CTC clusters) metastasis. CTC clusters represent a hybrid EMT, which indicates the presence of both epithelial (following cells) and mesenchymal cells (leader cells) in such clusters. Leader cells in the cluster show a mesenchymal phenotype, while most of the following cells represent an epithelial state (3).

Triple negative is the most aggressive breast cancer subtype (101), which represents high fraction of mesenchymal (M^+^) CTCs. High M^+^ CTC fraction is linked to the shorter progression-free survival (PFS) in patients with breast cancer (178). Targeting EMT can reduce the number of CTCs. Yang and colleagues in a phase 2 study evaluated the role of low-dose aspirin on metastatic CRC and noticed an increase in the number of epithelial-type (E^+^) CTCs while a decrease in the M^+^ CTC fraction (179). Liquid biopsy can be made for evaluation of M^+^ CTCs and E^+^ CTCs. In the metastatic breast cancer (178) and CRC (179), for instance, evaluation of M/E CTCs is considered as a well-established prognostic marker and a valuable tool for predicting responses to therapy. A point here is that monoclonal CTCs delineated by single cell migration of invasive tumor cells are more killed by NK cells compared with CTC clusters (3). To explain, cell-to-cell adhesion among the following cells in the CTC clusters reduces the expression of NK cell activating ligands (180). Therefore, for a tumor taking a metastatic phase it is suggested to address EMP instead of solo targeting of stemness.

### 7.4 Hypoxia-Inducible Factor 1

HIF-1 acts as a key role for CSC generation and maintenance (181). Hypoxia possibly retains CSCs in an undifferentiated state, and allowing only differentiation of cancer cells (182). Bulle and colleagues in a study evaluated the impact of the anti-septic drug acriflavine on xenograft pancreatic cancer. Acriflavine is drug that suppresses dimerization of HIF-1α and HIF-1β. Application of this drug for such a model considerably inhibited tumor growth only in a moderately differentiated cancer model, but not in a fast growing EMT ^high^ model (183).

## 8 Intra-tumoral Mesenchymal Stem Cells and Tumor Stemness

Generally, body organs tend to recall poorly differentiated cells upon encountering harmful conditions. This, in fact, serves as a compensatory mechanism to refill damaged cells. Mesenchymal stem cells (MSCs) from bone marrow are among the cells responding to signals rendered from injured organs. Normal MSCs recruited into the tumor area may transition into acquiring a CAF phenotype (184). One of the key roles of CAFs is the generation of supportive stem niches for protecting CSCs and promoting resistance (185).

## 9 Conclusions

The presence of immature cells in the tumor ecosystem is a turning point in tumor evolution, being important from diagnostic and therapeutic standpoints. Strategies for managing such potential in a tumor will open new therapeutic windows. Agents act on maturation of anti-tumor immune cells can thus be designed for strengthening the power of the immune system against cancer. IL-2, for instance, promotes maturation of T cells (186), and it can be a key component of most of the immunotherapeutic approaches (187) due to its effects on promoting the effector function of cells like macrophages, NK cells, and CD8^+^ T cells (59). A point of value here is that even when maturation occurs in a cell like DCs, it may not be sufficient to induce a strong immunity (18), so agents designed to act on maturation of anti-tumor immune cells must also induce an effector functionality. Another point to add is that maturity is not restricted to the tumor immunity. It can also include other cells or structures within a tumor. Tumor vasculature, for instance, is immature architecturally and functionally. Therefore, strategies can be expanded by inducing vascular maturity. A virtue of this approach is the more infiltration of anti-tumor immune cells, as well as anti-tumor drugs into the tumor area for promoting tumor killing activities. This will reduce immune escape and resistance and enhance responses to therapy.

## Author Contributions

KM gave the conceptualization. JM and KM wrote the initial manuscript. Final revisions were made by KM. Articles were selected by KM. Both authors approved the final draft.

## Conflict of Interest

The authors declare that the research was conducted in the absence of any commercial or financial relationships that could be construed as a potential conflict of interest.

## Publisher’s Note

All claims expressed in this article are solely those of the authors and do not necessarily represent those of their affiliated organizations, or those of the publisher, the editors and the reviewers. Any product that may be evaluated in this article, or claim that may be made by its manufacturer, is not guaranteed or endorsed by the publisher.
